# Roles of mitochondrial ROS and NLRP3 inflammasome in multiple ozone-induced lung inflammation and emphysema

**DOI:** 10.1186/s12931-018-0931-8

**Published:** 2018-11-22

**Authors:** Feng Li, Mengmeng Xu, Muyun Wang, Lei Wang, Hanying Wang, Hai Zhang, Yuqing Chen, Jicheng Gong, Junfeng(Jim) Zhang, Ian M. Adcock, Kian Fan Chung, Xin Zhou

**Affiliations:** 1 Department of Pulmonary Medicine, Shanghai Chest Hospital, Shanghai Jiaotong University, Shanghai, 200030 People’s Republic of China; 2 Department of Respiratory Medicine, Shanghai First People’s Hospital, Shanghai Jiaotong University, No.100, Haining Road, Shanghai, 200080 China; 3 Duke Global Health Institute and Nicholas School of the Environment, Duke University, Durham, NC 27708 USA; 4 College of Environmental Sciences and Engineering and BIC-ESAT, Peking University, Beijing, 100871 People’s Republic of China; 5 Global Health Research Center, Duke Kunshan University, Kunshan, Jiangsu 215316 People’s Republic of China; 6 Airway Disease Section, National Heart and Lung Institute, Imperial College London, London, SW3 6LY UK; 7 Priority Research Centre for Asthma and Respiratory Disease, Hunter Medical Research Institute, University of Newcastle, Newcastle, NSW 2305 Australia

**Keywords:** Mitochondrial ROS, NLRP3 inflammasome, Ozone, Lung inflammation, Emphysema

## Abstract

**Background:**

Mitochondrial damage leading to oxidant stress may play an important role in the pathogenesis of airflow obstruction and emphysema. NLPR3 inflammasome can be activated by mitochondrial ROS (mtROS) and other stimuli. We examined the importance of mtROS and NLRP3 inflammasome and their interactions in multiple ozone-induced lung inflammation and emphysema.

**Methods:**

C57/BL6 mice were exposed to ozone (2.5 ppm, 3 h) or filtered air twice a week over 6 weeks. MitoTEMPO (20 mg/kg), an inhibitor of mtROS, and VX765 (100 mg/kg), an inhibitor of caspase-1 activity, were administered by intraperitoneal or intragastric injection respectively 1 h prior to each ozone exposure for 6 weeks.

**Results:**

Ozone-exposed mice had increased bronchoalveolar lavage (BAL) total cells and levels of IL-1β, KC and IL-6, augmented lung tissue inflammation scores, enhanced oxidative stress with higher serum 8-OHdG concentrations, emphysema with greater mean linear intercept (Lm), airway remodeling with increased airway smooth muscle mass and airflow limitation as indicated by a reduction in the ratio of forced expiratory volume at 25 and 50 milliseconds to forced vital capacity (FEV_25_/FVC, FEV_50_/FVC). Both MitoTEMPO and VX765 reduced lung inflammation scores, cytokine levels, oxidative stress and increased mitochondrial fission proteins. VX765 also attenuated emphysema, airway remodeling and airflow limitation. MitoTEMPO inhibited the increased expression of mitochondrial complex II and IV and of NLPR3 while VX765 inhibited the expression and activity of NLRP3 and caspase-1 pathway in the lung.

**Conclusions:**

Both mtROS and NLRP3 inflammasome play a role in ozone-induced lung inflammation while only NLRP3 is involved in ozone-induced emphysema.

**Electronic supplementary material:**

The online version of this article (10.1186/s12931-018-0931-8) contains supplementary material, which is available to authorized users.

## Background

Chronic obstructive pulmonary disease (COPD) is a major lung disease currently affecting 384 million people worldwide, with a global prevalence of 11.7% [1]. The pathogenetic features of COPD, caused by cigarette smoke and other noxious particles or gases, include obstructive bronchiolitis and emphysema, which both lead to airflow limitation and respiratory symptoms [2]. The underlying mechanisms of COPD are not fully elucidated. However, oxidant-antioxidant imbalance or oxidative stress, due to over-production of reactive oxygen species (ROS) in excess of the antioxidant defenses, is a predominant mechanism for COPD [3].

Mitochondria are double membrane bound organelles that exist in most eukaryotic organisms. The morphology of mitochondria is regulated by fission and fusion. The former is mediated by dynamin related protein 1 (DRP1), Fission 1 (FIS1) and mitochondrial fission factor (MFF), while the latter is controlled by mitofusins 1 and 2 (MFN1 and MFN2), and optic atrophy protein 1 (OPA1) [4]. Mitochondria play an important role in production of adenosine triphosphate (ATP) and mitochondrial ROS (mtROS) [5]. Dysfunctional mitochondria influences airway contractility, gene expression, oxidative stress, immune response, cell proliferation, apoptosis and metabolism that are all implicated in COPD. Therefore, mitochondrial dysfunction is increasingly recognized as being involved in the pathogensis of COPD [6–8].

The nucleotide binding domain leucine-rich repeat-containing receptor (NLR) family members are cytosolic receptors that can sense pathogen- and damage -associated signaling, of which NLRP3 is the most widely characterized member. NLRP3 is proposed to play a role in the development of COPD [9]. Activation of the NLRP3 inflammasome involves a priming signal to upregulate the expression of NLRP3, and then the assembly of a multi-protein complex consisting of NLRP3 and pro-caspase-1, which enables caspase-1 activation and IL-1β maturation [10]. Mitochondrial dysfunction can also trigger NLRP3 activation, as supported by the co-localization of activated NLRP3 with mitochondria [11] and the finding that oxidized mitochondrial DNA is a direct ligand of NLRP3 [12]. In addition, other triggers such as lipopolysaccharide and danger signals such as ATP, hyaluronan and necrotic cells activate the NLPR3 inflammasome [13–15].

Ozone (O_3_) is an environmental pollutant that has been associated with worsening symptoms, increased hospitalizations and emergency visits in patients with COPD [16–18].We previously showed that repeated ozone exposure in mice led to chronic inflammation, emphysema and airflow limitation, all hallmark features of COPD [19, 20]. However, the molecular mechanisms of these effects are still not well established and an understanding of these may be relevant to the mechanisms underlying COPD. We hypothesize that NLRP3 inflammasome activation particularly by mtROS may be an important pathway in the development of COPD and therefore, inhibition of mtROS or of NLRP3 pathways may prevent ozone-induced features of COPD. We studied whether an mtROS inhibitor or a caspase-1 inhibitor could modulate the lung inflammation, emphysema, and airflow obstruction seen in the multiple ozone exposure mouse model.

## Methods

### Ozone exposure and inhibitor administration

**Fig. 1:**
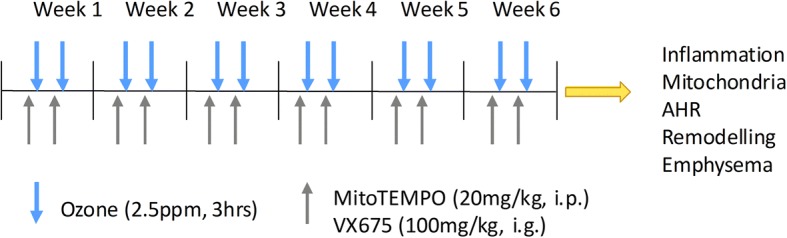
Schematic diagram of the experimental protocol. Mice were exposed to ozone(2.5 ppm) or filtered air for 3 h, twice a week over 6 weeks. Mice were intraperitoneally injected with PBS or MitoTEMPO (mtROS inhibitor, 20 mg/kg, dissolved in PBS) or orally fed by gavage with VX765 (caspase-1 inhibitor,100 mg/kg, dissolved in distilled water containing 0.5% sodium salt of carboxy methylellucose and 0.1% Tween-80) 1 hour before each exposure, twice a week for 6 weeks

The experimental procedures were approved by the Laboratory Animal Ethics Committee of Shanghai Chest Hospital. Eight-ten weeks male C57/BL6 mice (Shanghai Super- B&K Laboratory Animal Corp. Ltd., Shanghai, China) were housed in specific-pathogen-free (SPF) conditions with food and water supplied ad libitum. Mice were exposed to ozone (2.5 ppm) or filtered air for 3 h, twice a week over 6 weeks as previously described [19, 20]. Phosphate buffered saline (PBS, as vehicle) and MitoTEMPO (mtROS inhibitor, 20 mg/kg, Sigma-Aldrich, St. Louis, MO, USA) dissolved in PBS and administered intraperitoneally. VX765 (caspase-1 inhibitor,100 mg/kg, Selleck, Houston, TX, USA) was dissolved in distilled water containing 0.5% sodium salt of carboxy methylellucose and 0.1% Tween-80 and administered intragastrically to mice one hour before each exposure twice a week for 6 weeks. The detailed experimental protocol was outlined in Fig. 1.

To examine the effect of MitoTEMPO or VX765 on control mice, additional experiments were performed in which air-exposed mice were treated with 3 weeks of MitoTEMPO or VX765. An additional file shows this in more detail [see Additional file 1].

### Lung function

After anesthesia with injection of ketamine (100 mg/kg) and xylazine (10 mg/kg), mice were tracheostomized and placed in a whole-body plethysmograph (EMMS, Hants, UK) with a ventilator to measure inspiratory capacity (IC), functional residual capacity (FRC), total lung capacity (TLC), forced vital capacity (FVC), forced expiratory volume in first 25 and 50 milliseconds of exhalation (FEV_25_, FEV_50_) and chord compliance (Cchord) as previously described [19, 20].

### Measurement of bronchoalveolar lavage fluid and blood

Following terminal anaesthesia, bronchoalveolar lavage (BAL) fluid and blood were collected as previously described [19, 20]. Total cell counts were determined using a hemocytometer, and differential cell counts from cytospin preparations were measured under a microscope. BAL malondialdehyde (MDA) was measured using HPLC-fluorescence technique and serum 8-hydroxy-2′-deoxyguanosine (8-OHdG) was analyzed using HPLC-MS-MS technique as previously described [19, 20]. Levels of chemokine (C-X-C motif) ligand 1 (CXCL1, KC), interleukin-6 (IL-6) and interleukin-1β (IL-1β) in the BAL fluid were measured using enzyme-linked immunosorbent assay kits (R&D systems, China Co. Ltd., Shanghai, China) according to manufacturers’ instructions.

### Histological analysis

The left lung was inflated with 4% paraformaldehyde under 25 cm of water pressure and then embedded in paraffin. 5 μm sections were cut and stained with haematoxylin and eosin (H&E). The mean linear intercept (Lm), a measure of interalveolar septal wall distance, was determined using a reticule with 5 lines (each 500 μm long), with 10 fields per section assessed at random. Fields with airways or vessels were avoided. Lm was calculated by dividing the length of the line by the number of alveolar wall and grid line interceptions counted as described previously [19, 20].

Lung inflammatory response was scored on a 0–3 scale as previously described [20]: 0, no inflammation was detectable; 1, occasional cuffing with inflammatory cells; 2, most bronchi or vessels were surrounded by a thin layer (one to five cells thick) of inflammatory cells; 3, most bronchi or vessels were surrounded by a thick layer (more than five cells thick) of inflammatory cells. Lung inflammation was defined as the average of the peribronchial and perivascular inflammation scores.

To assess the change in the components of airway structure, the area of airway epithelium (Wae), airway smooth muscle (Wam), total airway wall (Wat), and the perimeter of basement membrane (Pbm) were measured in H&E-stained sections using a Motic digital microscope image analysis system (Motic China Group, Xiamen, Fujian, China) and parameters were calculated as Wae/Pbm, Wam/Pbm and Wat/Pbm as previously described [19, 20].

### Lung mtROS analysis and caspase-1 activity

Mitochondria were extracted from fresh lung tissues using a Mitochondria Isolation Kit for Tissue (Beyotime, Haimen, Jiangsu, China) with protease inhibitors using a Dounce tissue grinder following the manufacturer’s instructions as described before [21], and then quantified by BCA analysis (Thermo Fisher Scientific). Equal amounts of mitochondrial extract were incubated with 5 μM MitoSOX™ Red (Invitrogen) for 10 min at 37 °C and protected from the light. Red fluorescence was measured at 510/580 nm using a Flexstation®2 fluorescence reader (Molecular Devices, San Jose, CA, USA). The levels of caspase-1 activity in mouse lung tissue were detected using a commercial assay kit (Beyotime) and measured at 405 nm by a microplate reader following the manufacturer’s instructions as described before [22].

### Western blot

Equal amounts of mitochondrial extract or lung homogenate were separated by 10%SDS-PAGE (Beyotime) and electrophoretically transferred to nitrocellulose membranes. The membranes were blocked, then incubated with primary antibodies against total OXPHOS antibody cocktail (Abcam, Cambridge, MA, USA), voltage-dependent anion channel (VDAC, Abcam), DRP1 (Cell Signaling Technologies - CST, Beverly, MA, USA), MFF (CST), MFN2 (CST), OPA1 (CST), GAPDH (CST), NLRP3 (CST), caspase-1 (Abcam) and tubulin (CST) for blot detection. VDAC, GAPDH and tubulin were used as respective loading controls. The density was quantitated using a densitometer.

### Statistical analysis

All results were expressed as mean ± S.E.M. One-way analysis of variance (ANOVA) with Bonferroni post-test or Dunnett T3 post-test analysis performed for comparisons between multiple groups using SPSS 20.0 software. *P* < 0.05 was considered statistically significant.

## Results

### Lung function

**Fig. 2:**
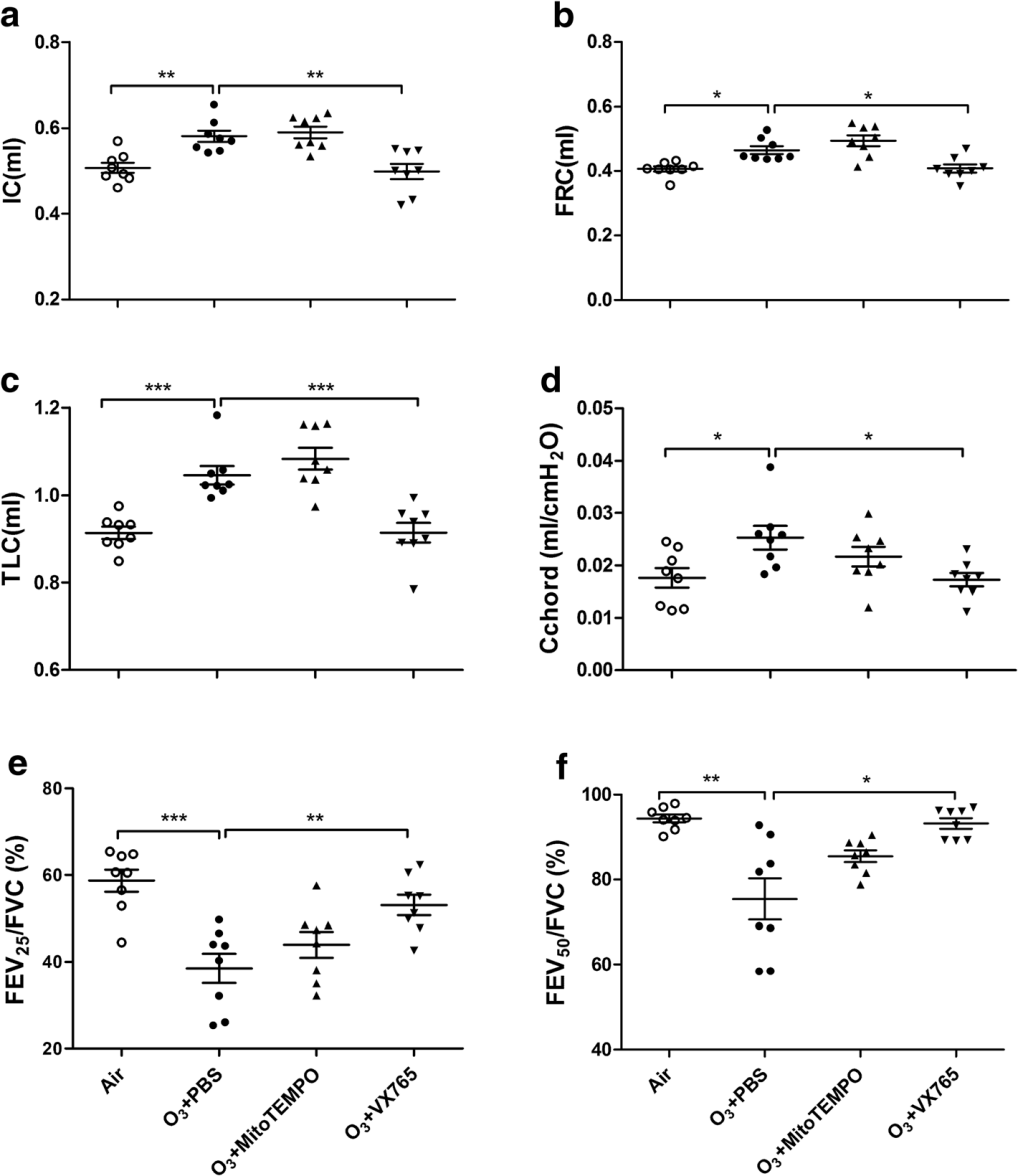
Effects of MitoTEMPO and VX765 on multiple ozone exposure-induced lung function changes. The top panel indicates the experimental plan. Drugs were delivered 1 h prior to ozone exposure. Lung function analysis was composed of inspiratory capacity (IC) (**a**), functional residual capacity (FRC) (**b**), total lung capacity (TLC) (**c**), chord compliance (Cchord) (**d**), and percentage of forced expiratory volume (FEV) in first 25 and 50 ms of fast expiration (FEV_25_ and FEV_50_) of forced vital capacity (FVC) (**e, f**). *P < 0.05, **P < 0.01, ***P < 0.001. One-way analysis of variance (ANOVA) with a Bonferroni or Dunnett’s T3 post-test analysis was performed for comparisons between multiple groups

Lung volume parameters (IC, FRC and TLC) and compliance (Cchord) were increased and airflow volume ratios (FEV_25_/FVC, FEV_50_/FVC) were decreased in ozone-exposed mice compared with control mice. Treatment with VX765 inhibited the increase in IC, FRC, TLC and Cchord, and prevented the decrease in FEV_25_/FVC and FEV_50_/FVC, while treatment with MitoTEMPO showed no significant effect on any of these parameters (Fig. 2a-f).

### BAL fluid cells and cytokines and lung inflammatory socres

**Fig. 3:**
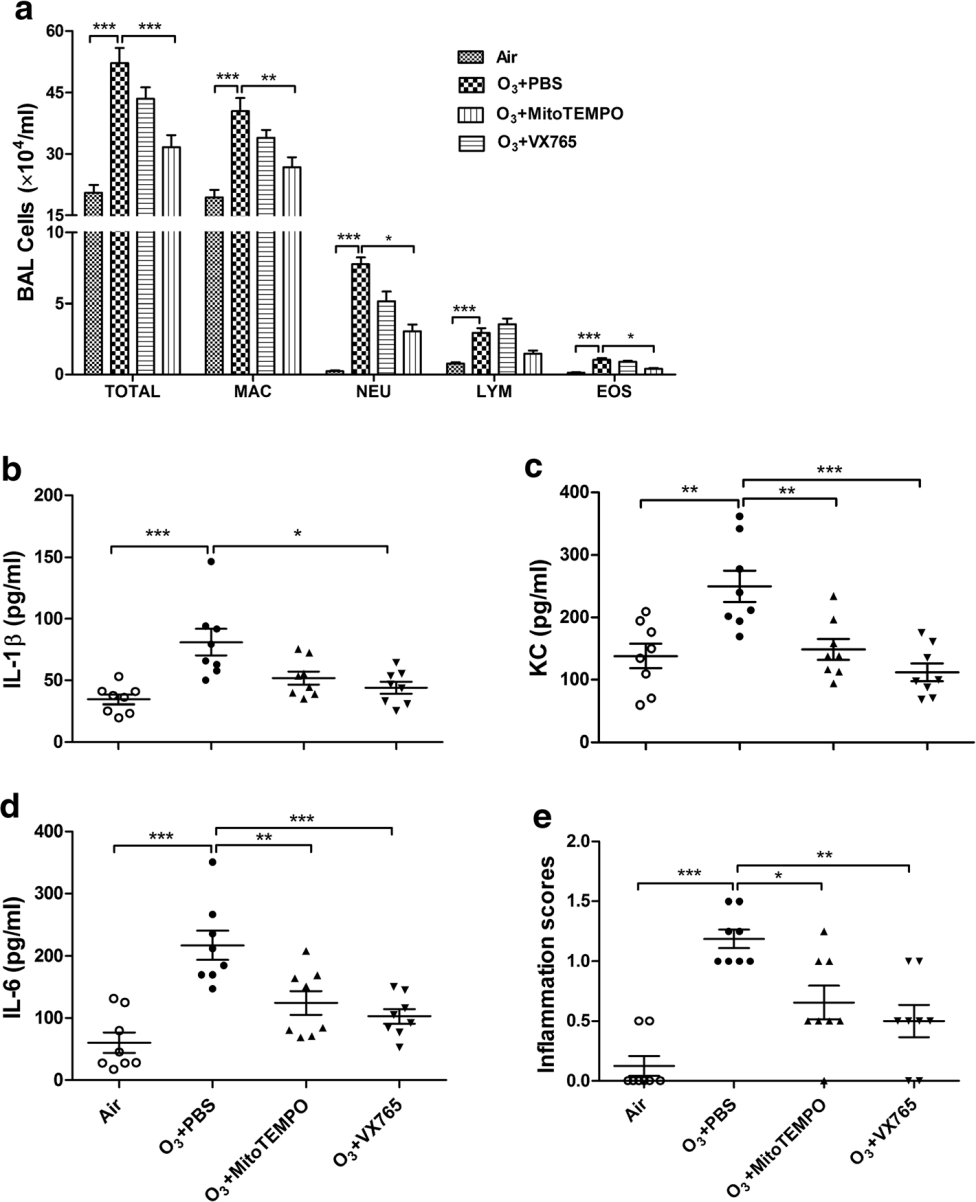
Effects of MitoTEMPO and VX765 on multiple ozone exposure-induced airway inflammation and and lung inflammation. **a** Effects of MitoTEMPO and VX765 on multiple ozone exposure-induced increased cell numbers in bronchoalveolar lavage (BAL) fluid, including total cells (TOTAL), macrophages (MAC), lymphocytes (LYM), neutrophils (NEU) and eosinophils (EOS). Each bar represents the mean ± SEM. **b-d** Effects of MitoTEMPO and VX765 on multiple ozone exposure-induced increased levels of cytokines in BAL fluid, including IL-1β (**b**), KC (**c**) and IL-6 (**d**). **e** Effects of MitoTEMPO and VX765 on multiple ozone exposure-induced inflammation scores in lung tissue. *P < 0.05, **P < 0.01, ***P < 0.001. One-way analysis of variance (ANOVA) with Bonferroni or Dunnett’s T3 post-test analysis was performed for comparisons between multiple groups

Total BAL cell counts, including macrophages, lymphocytes, neutrophils and eosinophils, were increased in ozone-exposed mice compared with control mice. Treatment with VX765 decreased the numbers of total cells, macrophages, neutrophils and eosinophils. In contrast, treatment with MitoTEMPO did not affect total or differential cell counts although there was a trend towards reduced counts except for lymphocytes (Fig. 3a).

Increased lung inflammation scores with inflammatory cell infiltrates around bronchi or vessels in lung sections were observed in ozone-exposed mice compared with control mice. Treatment with MitoTEMPO and VX765 reduced lung inflammation scores (Fig. 3b).

Increases in the level of IL-1β, KC and IL-6 were observed in ozone-exposed mice compared to control mice. MitoTEMPO treatment caused a reduction in the levels of KC and IL-6 and had a non-significant reduction in IL-1β concentration. VX765 treatment significantly decreased the levels of KC, IL-6 and IL-1β (Fig. 3c-e).

### Oxidative stress and lung caspase-1 activity

**Fig. 4:**
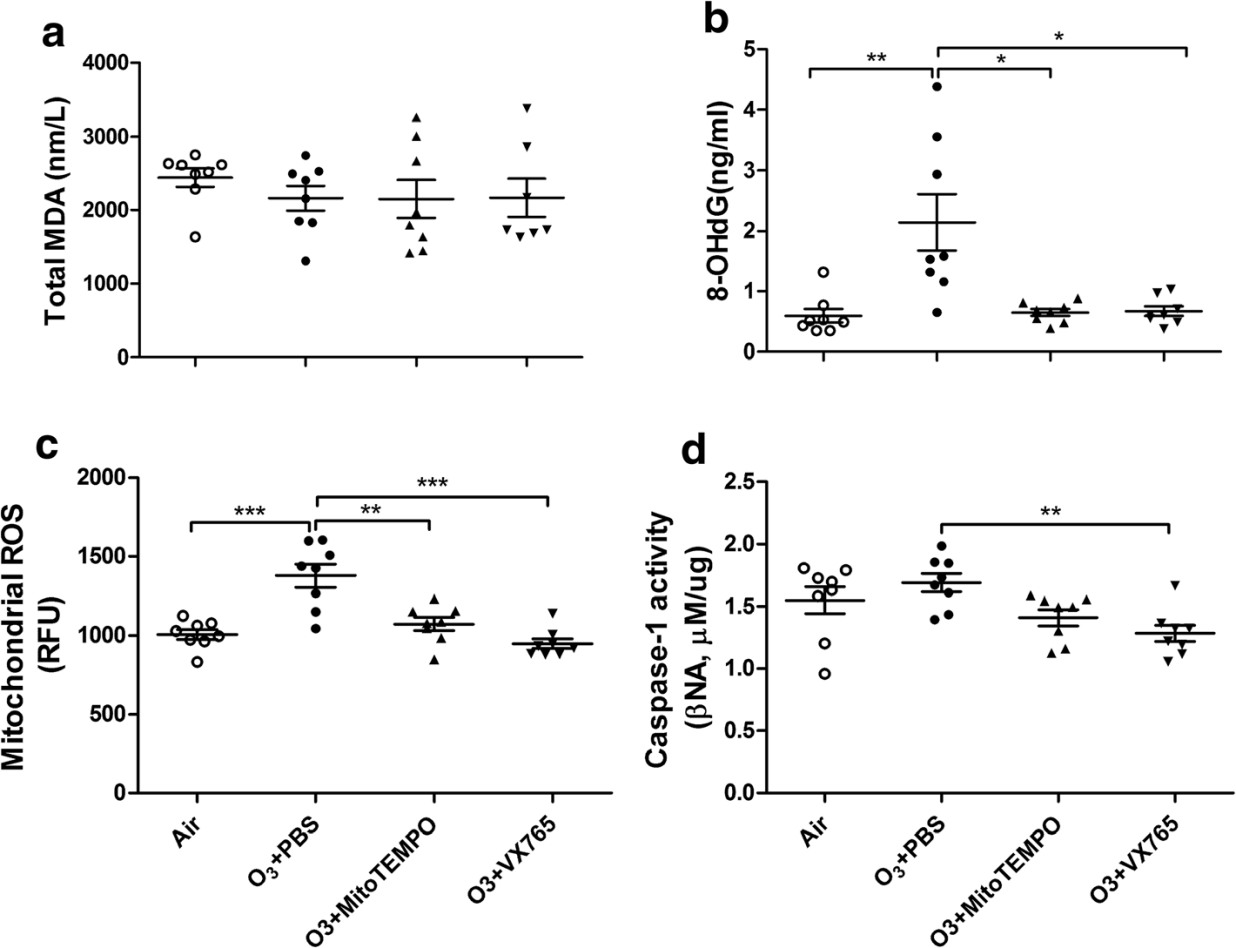
Effects of MitoTEMPO and VX765 on multiple ozone exposure-induced increases in malonaldehyde in BAL fluid (**a**), 8-hydroxy-deoxyguanosine (8-OHdG) in serum (**b**), mitochodrial ROS (**c**) and caspase-1 activity (**d**) in lung tissue. *P < 0.05, **P < 0.01, ***P < 0.001. One-way analysis of variance (ANOVA) with Bonferroni or Dunnett’s T3 post-test analysis was performed for comparisons between multiple groups

There was no increase in BAL MDA in ozone-exposed mice compared to control mice. Neither MitoTEMPO nor VX765 affected MDA concentrations (Fig. 4a). In contrast, levels of serum 8-OHdG were increased in ozone-exposed mice compared to control mice. MitoTEMPO and VX765 intervention both reduced serum 8-OHdG levels (Fig. 4b). Serum 8-OHdG was negatively correlated with FEV_25_/FVC (*r* = − 0.4021, *P* < 0.05), FEV_50_/FVC (*r* = − 0.3815, P < 0.05), and positively correlated with BAL total cells (*r* = 0.4283, *P* < 0.05), macrophages (*r* = 0.4221, P < 0.05), neutrophils (*r* = 0.4462, P < 0.05), eosinophils (*r* = 0.4494, *P* < 0.01), IL-1β (*r* = 0.5382, P < 0.01), KC (*r* = 0.5743, *P* < 0.001) and IL-6 (*r* = 0.4147, *P* < 0.05).

Increased mtROS in lung was found in ozone-exposed mice compared to control mice. Treatment with MitoTEMPO and VX765 reduced mtROS (Fig. 4c). Lung mtROS levels were positively correlated with 8-OHdG (*r* = 0.4672, *P* < 0.01) and negatively correlated with FEV25/FVC (*r* = − 0.4469, *P* < 0.05), FEV50/FVC (*r* = − 0.5537, P < 0.01). Lung mtROS also positively correlated with BAL total cells (*r* = 0.4534, P < 0.01), macrophages (*r* = 0.4178, P < 0.05), neutrophils (*r* = 0.5622, *P* < 0.001), eosinophils (*r* = 0.4229, P < 0.05), IL-1β (*r* = 0.4499, P < 0.01), KC (*r* = 0.5996, P < 0.001) and IL-6 (*r* = 0.4347, P < 0.05),

There was no difference in caspase-1 activity in ozone-exposed mice compared to control mice. Treatment with VX765 reduced caspase-1 activity, while treatment with MitoTEMPO showed no effect (Fig. 4d).

### Histological analysis

**Fig. 5:**
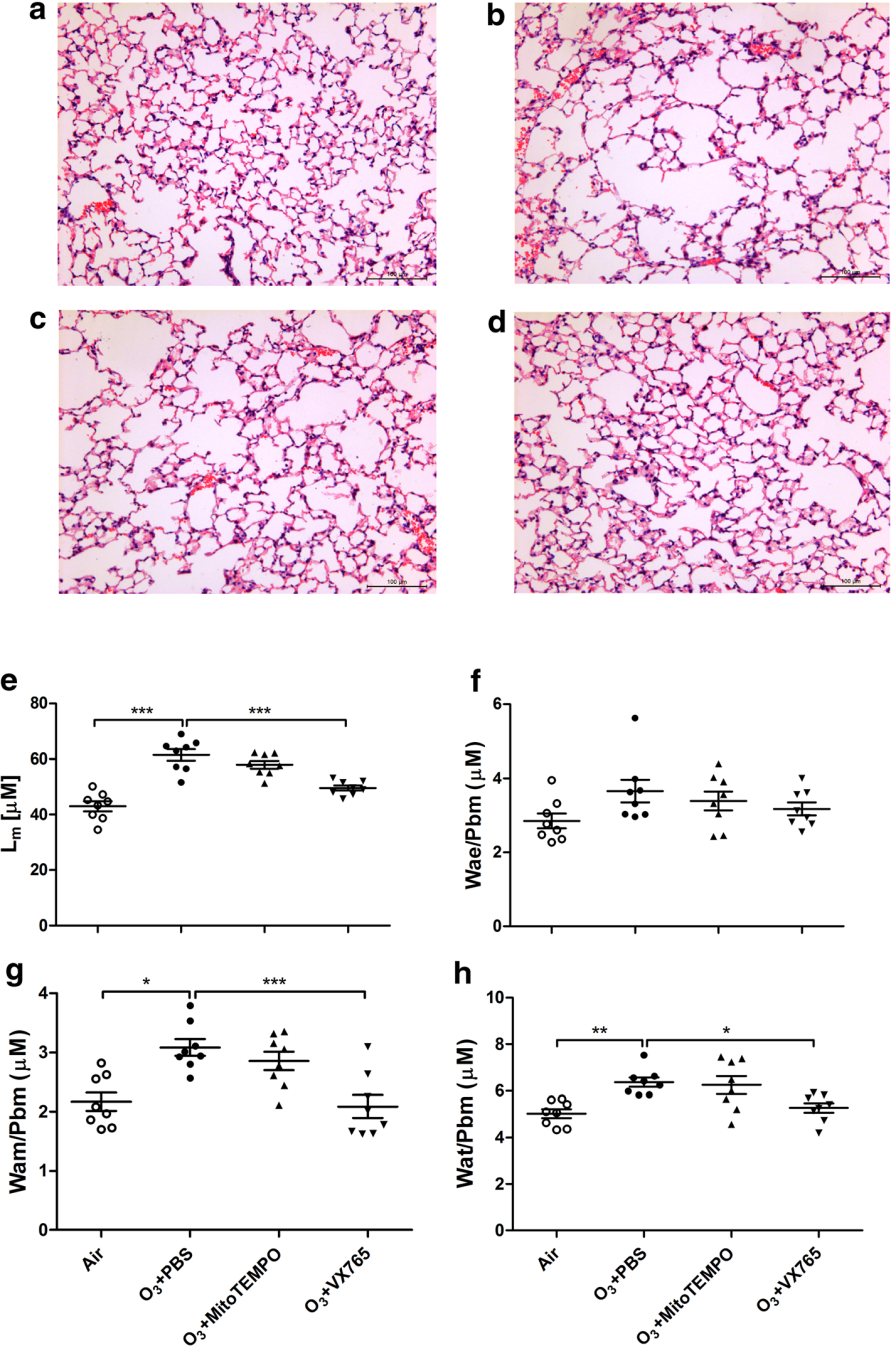
Representative photomicrographs of lung alveolar spacesin haematoxylin-eosin-stained sections of air-exposed mice (**a**),ozone-exposed mice (**b**), MitoTEMPO-treated ozone-exposed mice (**c**), VX765-treated ozone exposed mice(**d**). (bar = 100 μm). Effects of MitoTEMPO and VX765 on multiple ozone exposure-induced increase in mean linear intercept (L_m_) (**e**) and changes in airway structure including thickness of airway epithelial layer (Wae/Pbm) (**f**), airway smooth muscle (Wam/Pbm) (**g**) and total airway wall (**h**). *P < 0.05, **P < 0.01, *** *P* < 0.001. One-way analysis of variance (ANOVA) with Bonferroni or Dunnett’s T3 post-test analysis was performed for comparisons between multiple groups

Lung alveolar enlargement after ozone exposure is depicted in Fig. 5a–d. There were increases in Lm, Wam and Wat in ozone-exposed mice compared with control mice. Treatment with VX765 prevented the increases of Lm, ASM and Wat in ozone-exposed mice. However, MitoTEMPO treatment affected none of these histological parameters (Fig. 5e, g, h). Lm was positively correlated with 8-OHdG (*r* = 0.5407, *P* < 0.01) and mtROS (*r* = 0.4288, *P* < 0.01). In addition, Wam/Pbm (*r* = 0.5859, *P* < 0.001) and Wat/Pbm (*r* = 0.4610, *P* < 0.01) were positively correlated with mtROS.

### Expression of mitochondrial OXPHOS, mitochondria-related proteins, NLRP3 & caspase-1

**Fig. 6:**
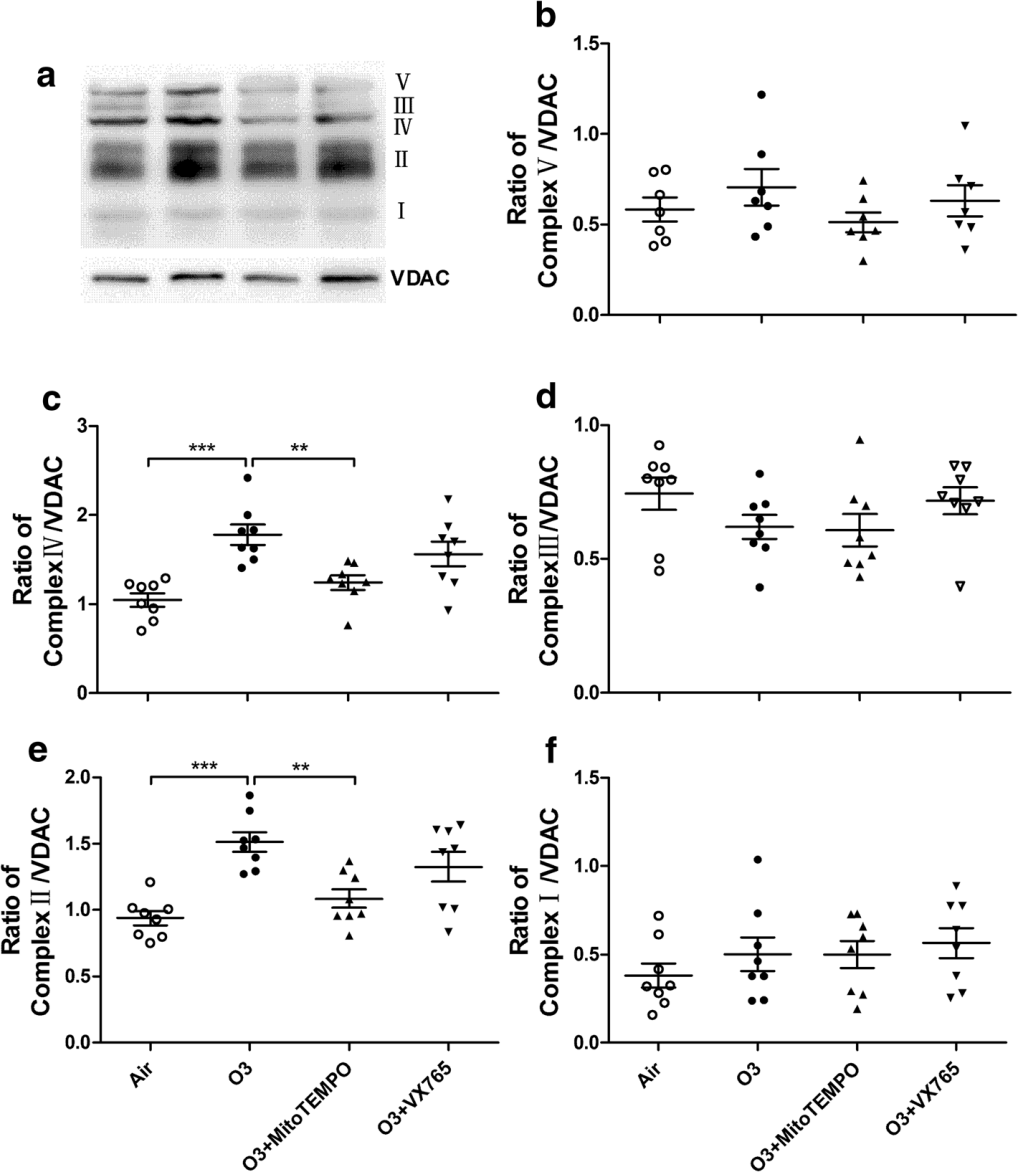
Representative Western blot images of mitochondrial OXPHOS and VDAC in murine lung tissue. (**a**) Effects of MitoTEMPO and VX765 on the protein expression of OXPHOS complex V (**b**), IV (**c**), III(**d**), II (e) and I (**f**) in the lung after multiple ozone exposure.^*^P < 0.05, ^**^P < 0.01, ^***^ P < 0.001. One-way analysis of variance (ANOVA) with Bonferroni or Dunnett’s T3 post-test analysis was performed for comparisons between multiple groups

There was increased expression of mitochondrial OXPHOS complexes II and IV in ozone-exposed mice compared with control mice. MitoTEMPO treatment inhibited the expression of mitochondrial complex II and IV in the lung tissue whilst VX765 treatment showed no effect (Fig. 6a, d, e). Mitochondrial complex II expression was positively correlated with mtROS (*r* = 0.4466, *P* < 0.05), and mitochondrial complex IV expression was positively correlated with 8-OHdG (*r* = 0.4062, *P* < 0.05).

**Fig. 7:**
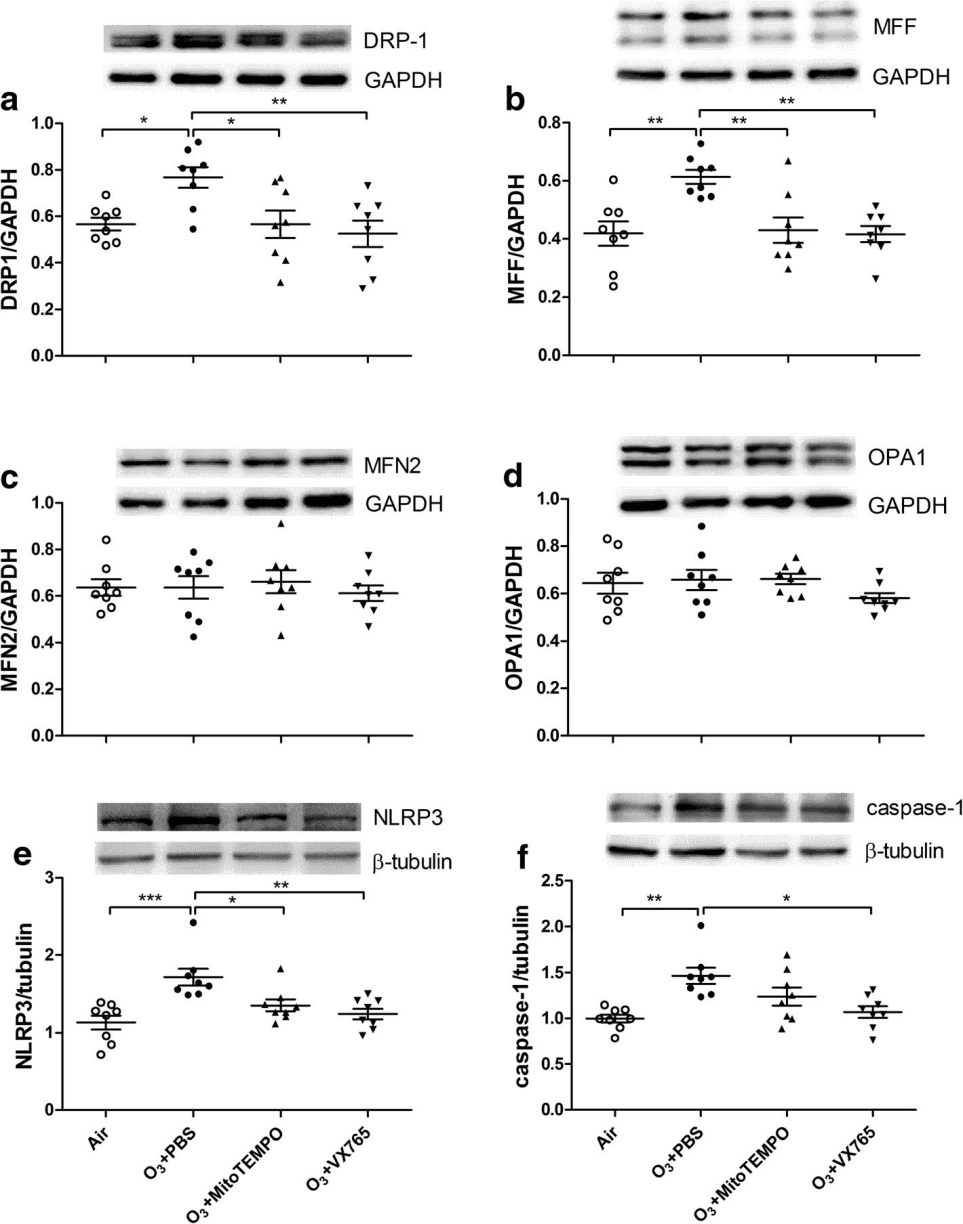
Effects of MitoTEMPO and VX765 on the protein expression of mitochondrial fission/fusion-related proteins including DRP1 (**a**), MFF (**b**), MFN2 (**c**) and OPA1 (**d**), and NLRP3 (**e**) and caspse-1 (**f**) in the lung after multiple ozone exposure. Each panel shows representative Western blot. ^*^P < 0.05, ^**^P < 0.01, ^***^ P < 0.001. One-way analysis of variance (ANOVA) with Bonferroni or Dunnett’s T3 post-test analysis was performed for comparisons between multiple groups

The expression of DRP1 and MFF was increased in ozone-exposed mice compared to control mice and both MitoTEMPO and VX765 treatment inhibited the expression of DRP1 and MFF (Fig. 7a, b). The expression of MFN2 and OPA1 was unchanged in ozone-exposed mice compared to control mice and neither MitoTEMPO nor VX765 affected MFN2 or OPA1 expression (Fig. 7c, d). DRP1 expression was positively correlated with 8-OHdG (*r* = 0.4938, *P* < 0.01) and mtROS (*r* = 0.3498, P < 0.05) whilst MFF expression was positively correlated with 8-OHdG (*r* = 0.5319, *P* < 0.01) and mtROS (*r* = 0.4319, *P* < 0.05).

The expression of NLPR3 and caspase-1 was increased in ozone-exposed mice compared to control mice. MitoTEMPO intervention inhibited the expression of NLRP3, while VX765 treatment inhibited the expression of NLPR3 and caspase-1 (Fig. 7e, f). NLRP3 expression was positively correlated with 8-OHdG (*r* = 0.4081, *P* < 0.01) and mtROS (*r* = 0.5772, *P* < 0.001), and caspase-1 expression was positively correlated with 8-OHdG (*r* = 0.6376, P < 0.001) and mtROS (*r* = 0.4929, *P* < 0.01).

The overall effects of MitoTEMPO and VX-765 on ozone-induced inflammation, lung function and emphysema are summarized in Table 1.

**Table 1:** The effects of MitoTEMPO and VX-765 on ozone-induced lung inflammation and emphysema

Parameter	MitoTEMPO	VX-765
IC, FRC, TLC, Cchord	NE	↓
FEV_25_/FVC, FEV_50_/FVC	NE	↑
Bronchoalveolar lavage cells	NE	↓total cells,macrophages, neutrophils,eosinophils
Lung inflammation score	↓	↓
Inflammatory cytokines	↓KC, IL-6	↓IL-1β, KC, IL-6
Oxidative stress	↓8-OHdG, lung mtROS	↓8-OHdG,lung mtROS
Mean linear intercept	NE	↓
Airway structure components	NE	↓Wam/Pbm, Wat/Pbm
Mitochondrial OXPHOS	↓complex II,IV	NE
Mitochondrial fission protein	↓DRP1,MFF	↓DRP1,MFF
Mitochondrial fusion protein	NE	NE
NLRP3 protein	↓	↓
Caspase-1 protein	NE	↓
Caspase-1 activity	NE	↓

The effects of MitoTEMPO and VX-765 on lung function,lung inflammation (bronchoalveolar lavage cells, inflammation score,Inflammatory cytokines), oxidative stress, lung histology(mean linear intercept, airway structure components), mitochondrial changes (OXPHOS, fission- and fusion-related proteins) and expression and activity of NLRP3/caspase-1

*NE* no effect ↓decrease ↑increase

## Discussion

In the present study, both MitoTEMPO and VX765 reduced multiple ozone-induced features including lung inflammation, oxidative stress, and increased mitochondrial fission proteins. In addition, VX765 reduced emphysema, airway remodeling and airflow limitation that were not affected by MitoTEMPO. MitoTEMPO specifically inhibited the expression of mitochondrial complexes II and IV and of NLRP3, while VX765 inhibited the expression and activity of NLRP3-caspase-1 pathway in the lung tissue. These data suggest that both mitochondrial dysfunction and NLRP3 activation may be involved in the pathogenesis of COPD. Targeting mtROS ameliorated some, but not all, of the features modulated by inhibition of the downstream target NLRP3 inflammasome/caspase 1. The data suggests that mtROS may not activate NLRP3 inflammasome-mediated ozone-induced emphysema.

Consistent with previous studies [19, 20], our experiments confirmed that multiple ozone exposure caused lung inflammation. This was reflected by increased numbers of total cells, macrophages, lymphocytes, neutrophils and eosinophils, and increased levels of KC, IL-6 and IL-1β in BAL fluid, along with increased inflammatory scores in lung sections after ozone exposure. In our experiments, the dose of MitoTEMPO and VX765 was chosen according to previous studies [23, 24] and our own pilot data. MitoTEMPO failed to inhibit the recruitment of inflammatory cells following ozone exposure, but reduced KC and IL-6 levels in BAL fluid and inflammation scores in lung tissue. In contrast, VX765 reduced ozone effects on inflammatory cells, KC, IL-6 and IL-1β in BAL fluid and inflammation scores in lung tissue. These findings indicate that both mtROS and NLRP3 played an important role in ozone-induced lung inflammation.

Ozone is a strong oxidizing agent, and can generate ROS and oxidative stress, which is a major mechanism in ozone-induced lung injury. Consistent with our previous study [20], the present study showed that multiple ozone exposure induced an increase in serum 8-OHdG, but not in BAL MDA. A recent study showed that increased mtROS was observed in lung macrophages after 72 h of 0.7 ppm ozone exposure [25]. We here demonstrated that elevated mtROS levels in lung tissue of mouse by multiple ozone exposure. MitoTEMPO treatment reduced mtROS in cigarette smoke extract (CSE)-treated human lung fibroblast cells [26]. Our results showed that MitoTEMPO reduced 8-OHdG in serum and mtROS in lung tissue. Mitochondrial dysfunction including mtROS has been proposed to stimulate NLRP3 inflammasome activation [27] and activated NLRP3 inflammasome in turn may lead to mitochondrial damage [28]. In the present study, we observed that VX765 reduced serum 8-OHdG and lung mtROS, which could occur through inhibition of oxidative stress as a result of suppressing the activation of NLRP3 inflammasome.

Multiple ozone exposure-induced emphysema was confirmed by the increased mean linear intercept (Lm) in the lung sections, which indicates an increase in alveolar size. Further analysis showed that airway smooth muscle layer and total airway wall were increased in the ozone-exposed mice, compatible with the airway remodeling seen in patients with COPD [29]. In line with these, there were increases in the lung volume parameters including IC, FRC and TLC and in lung compliance (Cchord), and decreases in the airflow as indicated by decreases in the ratios of FEV_25_/FVC and FEV_50_/FVC in ozone-exposed mice. We observed that treatment with VX765, but not MitoTEMPO, prevented the development of emphysema, airway remodeling and airflow limitation in ozone-exposed mice. We interpreted these results as indicating that NLPR3 activation is through other mechanisms apart from mtROS as far as the development of these ozone-induced features is concerned.

Mitochondrial dysfunction including increased mtROS production and impaired OXPHOS has been linked to the pathogenesis of COPD [30]. The expression of mitochondrial OXPHOS complexes was increased in BEAS-2B cells by long term CSE exposure as well as in bronchial epithelial cells from COPD patients [31]. The increased expression of mitochondrial complex II and IV in ozone-exposed mice may indicate increased capacity of OXPHOS to produce mtROS, as the treatment with MitoTEMPO inhibited the expression of both complexes as well as the levels of mtROS.

Mitochondria are regulated through cycles of fusion and fission. Mitochondrial fussion is induced by acute oxidative stress, which possibly acts as defense against ROS, while mitochondrial fission is induced by more prolonged oxidative stress and mitochondrial dysfunction, which facilitates removal of damaged mitochondria and induction of apoptosis [7]. Increased expression of DRP1 was found in a mouse emphysema model induced by over-expression of cathepsin E where inhibition of DRP1 prevented cell apoptosis and emphysema [32]. Similarly, in the present study, mitochondrial fusion-related proteins were unchanged whilst mitochondrial fission-related proteins were enhanced. This indicates that increased mitochondrial fission occurs in the lung tissue of ozone-induced lung inflammation and emphysema.

The activity of NLRP3 was elevated in various lung inflammation models. For example, IL-1β, IL-6 and TNF-α were elevated in BAL fluid and NLRP3 was activated in lung tissue of lipopolysaccharide(LPS)-induced lung injury model [33]. IL-1β concentrations in BAL fluid and NLRP3 mRNA in lung tissue were increased in PM_2.5_-induced acute lung inflammation model [34]. Most animal models have shown a crucial role for the NLRP3 inflammasome in the inflammatory and immune responses in COPD. However, the data in humans have been more variable [35]. A recent study using an anti-IL-1R mAb (MED18968) was ineffective in COPD [36]. More long-term clinical trials with canakinumab or NLRP3-specific agents are needed [37]. The mtROS inhibitor NecroX-5 reduces NLRP3 inflammasome activation in an allergic mouse model [38]. Our study showed that MitoTEMPO inhibited the expression of NLRP3 in lung tissue, which may indicate that mtROS is the upstream of NLRP3 and that inhibition of mtROS can lead to a reduction of NLRP3 expression. The present study also showed that VX765 inhibited the expression of both NLRP3 and caspase-1 in lung tissue.

## Conclusions

In summary, these results from our study support our hypothesis that mtROS and NLRP3 inflammasome are involved in ozone-induced lung inflammation, while only NLRP3 inflammasome is involved in ozone-induced lung emphysema. This suggests that the latter is independent of mtROS and may be activated by non-mtROS mechanisms.

## Additional file

Additional file 1: The effect of 3-week treatment with MitoTEMPO or VX765 on air-exposed mice. (DOC 5569 kb)

## Abbreviations

8-OHdG: 8-hydroxy-2′-deoxyguanosine
ATP: Adenosine triphosphate
BAL: Bronchoalveolar lavage
Cchord: Chord compliance
COPD: Chronic obstructive pulmonary disease
CSE: Cigarette smoke extract
CXCL1: Chemokine (C-X-C motif) ligand 1
DRP1: Dynamin related protein
FEV_25_: Forced expiratory volume at 25 milliseconds
FEV_50_: Forced expiratory volume at 50 milliseconds
FRC: Functional residual capacity
FVC: Forced vital capacity
IC: Inspiratory capacity
IL-1β: Interleukin-1β
IL-6: Interleukin-6
LPS: Lipopolysaccharide
MDA: Malondialdehyde
MFF: Mitochondrial fission factor
mtROS: Mitochondrial ROS
NE: No effect
NLR: Nucleotide binding domain leucine-rich repeat-containing receptor
OPA1: Optic atrophy protein 1
PBS: Phosphate buffered saline
ROS: Reactive oxygen species
TLC: Total lung capacity
