# Supplementary material for: Roles of mitochondrial ROS and NLRP3 inflammasome in multiple ozone-induced lung inflammation and emphysema
Source: Respir Res. 2018 Nov 22;19:230. doi: 10.1186/s12931-018-0931-8 (PMC6249848; doi:10.1186/s12931-018-0931-8)
Supplement: Supplementary file 1 — The effect of 3-week treatment with MitoTEMPO or VX765 on air-exposed mice. (DOC 5569 kb) [file 12931_2018_931_MOESM1_ESM.doc]

**Additional file 1**

**Roles of mitochondrial ROS and NLRP3 inflammasome in multiple ozone-induced lung inflammation and emphysema**

Feng Li^1,2^*, Mengmeng Xu^1^*,Muyun Wang^1^*, Lei Wang^2^, Hanying Wang^2^, Hai Zhang^1^,Yuqing Chen^1^, Jicheng Gong^3,4^, Junfeng (Jim) Zhang^3,5^, Ian M. Adcock^5,6^, Kian Fan Chung^5^, Xin Zhou^1＃^

The effect of MitoTEMPO and VX765 on lung function, lung inflammatory response, and expression and activity of NLRP3 inflammasome

**Methods**

**mice and inhibitor administration**

Eight-ten weeks male C57/BL6 mice (Shanghai Super- B&K Laboratory Animal Corp. Ltd, Shanghai, China) were housed in specific-pathogen-free (SPF) conditions with food and water supplied ad libitum. Mice were exposed filtered air for 3 hours, twice a week over 3 weeks. Phosphate buffered saline (PBS) and MitoTEMPO (mtROS inhibitor, 20mg/kg, Sigma-Aldrich, St. Louis, MO, USA) dissolved in PBS and administered intraperitoneally to mice, while VX765 (caspase-1 inhibitor,100mg/kg, Selleck, Houston, TX, USA) was dissolved in distilled water containing 0.5% sodium salt of carboxy methylellucose and 0.1% Tween-80 and administered intragastrically to mice one hour before each exposure twice a week for 3 weeks.

**Lung function**

After anesthesia with injection of ketamine (100mg/kg) and xylazine (10mg/kg) , mice were tracheostomized and placed in a whole-body plethysmograph (EMMS, Hants, UK) with a ventilator to measure inspiratory capacity (IC), functional residual capacity (FRC), total lung capacity (TLC), forced vital capacity (FVC), forced expiratory volume in first 25 and 50 milliseconds of exhalation (FEV_25_, FEV_50_) and chord compliance (Cchord).

**Measurement of bronchoalveolar lavage fluid**

Following terminal anaesthesia, bronchoalveolar lavage (BAL) fluid collected. Total cell counts were determined using a hemocytometer, and differential cell counts from cytospin preparations were measured under a microscope. Levels of chemokine (C-X-C motif) ligand 1 (CXCL1, KC), interleukin-6 (IL-6) and interleukin-1β (IL-1β) in the BAL fluid were measured using enzyme-linked immunosorbent assay kits (R&D systems, China Co. Ltd, Shanghai, China) according to manufacturers' instructions.

**Histological analysis**

The left lung was inflated with 4% paraformaldehyde under 25cm of water pressure and then embedded in paraffin. 5µm sections were cut and stained with haematoxylin and eosin (H&E).Lung inflammatory response was scored on a 0–3 scale as previously described: 0, no inflammation was detectable; 1, occasional cuffing with inflammatory cells; 2, most bronchi or vessels were surrounded by a thin layer (one to five cells thick) of inflammatory cells; 3, most bronchi or vessels were surrounded by a thick layer (more than five cells thick) of inflammatory cells. Lung inflammation was defined as the average of the peribronchial and perivascular inflammation scores.

**Lung caspase-1 activity**

The levels of caspase-1 activity in mouse lung tissue were detected using a commercial assay kit (Beyotime) and measured at 405 nm by a microplate reader following the manufacturer’s instructions.

**Western blot**

Equal amounts lung homogenate were separated by 10%SDS-PAGE (Beyotime) and electrophoretically transferred to nitrocellulose membranes. The membranes were blocked, then incubated with primary antibodies against NLRP3 (CST), caspase-1 (Abcam) and GAPDH (CST) for blot detection. The density was quantitated using a densitometer.

Results:

1. Lung Function

Compared with PBS-treated air-exposed mice, treatment with MitoTEMPO or VX765 for 3 weeks did not significantly affect lung volume parameters (IC, FRC and TLC), compliance (Cchord) or airflow volume ratios (FEV_25_/FVC, FEV_50_/FVC) .

**

**

2. BAL cells, cytokines and inflammation scores

Compared with PBS-treated air-exposed mice, treatment with MitoTEMPO or VX765 for 3 weeks did not significantly affect total cells and differential cell counts, or cytokines levels in BAL fluid and lung inflammation scores in air-exposed mice.





3. NLRP3, Caspase-1 expression and caspase-1 activity

Compared with PBS-treated air-exposed mice, treatment with MitoTEMPO or VX765 for 3 weeks did not significantly affect the expression of NLRP3 and caspase-1, or capase-1 activity in air-exposed mice.

**
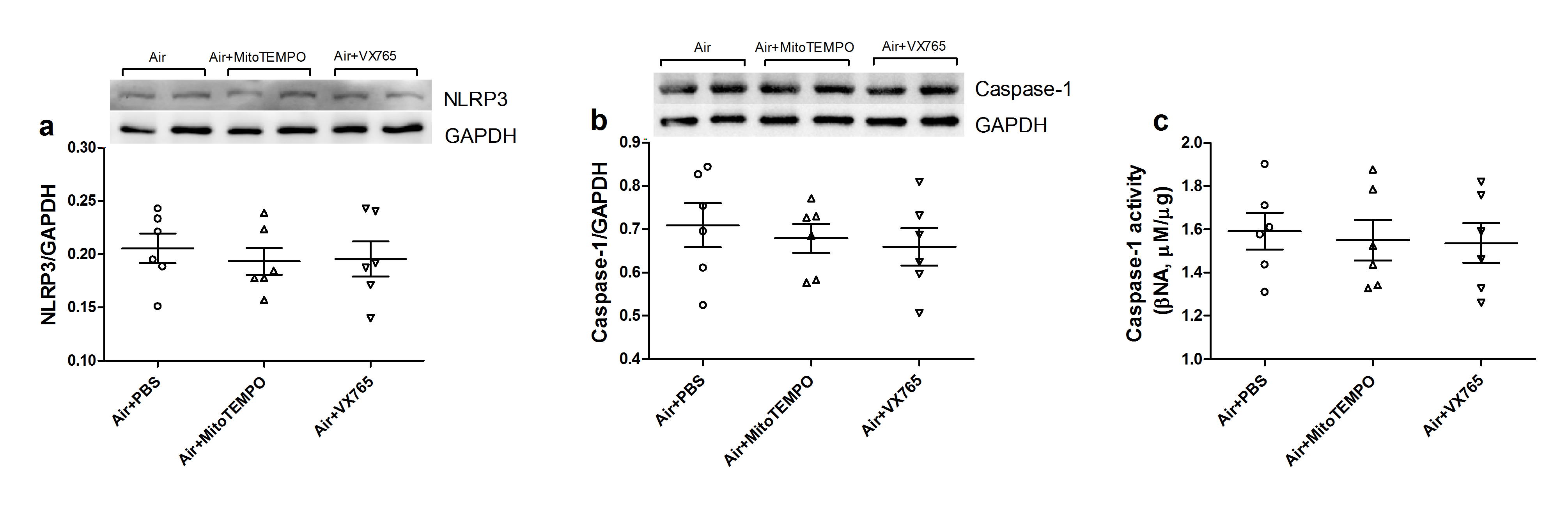
**
